# Accessing Local Knowledge to Identify Where Species of Conservation Concern Occur in a Tropical Forest Landscape

**DOI:** 10.1007/s00267-013-0051-7

**Published:** 2013-05-01

**Authors:** Michael Padmanaba, Douglas Sheil, Imam Basuki, Nining Liswanti

**Affiliations:** 1 Center for International Forestry Research, P.O. Box 0113 BOCBD, Bogor, 16000 Indonesia; 2 School of Environment, Science and Engineering, Southern Cross University, PO Box 157, Lismore, NSW 2480 Australia; 3 Institute of Tropical Forest Conservation (ITFC), Mbarara University of Science and Technology (MUST), P.O. Box 44, Kabale, Uganda; 4 JL. CIFOR, Situ Gede, Sindang Barang, Bogor, 16115 Indonesia

**Keywords:** Local knowledge, Accuracy, Expertise time and budget, Conservation, East Kalimantan

## Abstract

Conventional biodiversity surveys play an important role in ensuring good conservation friendly management in tropical forest regions but are demanding in terms of expertise, time, and budget. Can local people help? Here, we illustrate how local knowledge can support low cost conservation surveys. We worked in the Malinau watershed, East Kalimantan, Indonesia, an area currently at risk of extensive forest loss. We selected eight species of regional conservation interest: rafflesia (*Rafflesia* spp.), black orchid (*Coelogyne pandurata*), sun bear (*Helarctos malayanus*), tarsier (*Tarsius bancanus*), slow loris (*Nycticebus coucang*), proboscis monkey (*Nasalis larvatus*), clouded leopard (*Neofelis diardi/N. nebulosa*), and orang-utan (*Pongo pygmaeus*). We asked 52 informants in seven villages if, where and when they had observed these species. We used maps, based on both geo-referenced and sketched features, to record these observations. Verification concerns and related issues are discussed. Evaluations suggest our local information is reliable. Our study took 6 weeks and cost about USD 5000. Extensive expert based field surveys across the same region would cost one or two orders of magnitude more. The records extend the known distribution for sun bear, tarsier, slow loris, and clouded leopard. Reports of rafflesia, proboscis monkey, and orang-utan are of immediate conservation significance. While quality concerns should never be abandoned, we conclude that local people can help expand our knowledge of large areas in an effective, reliable, and low cost manner and thus contribute to improved management.

## Introduction

Because we cannot expect managers to control and protect all species that occur in tropical forests, we need effective priorities. But to set priorities managers need good information on where species of conservation significance occur. Across much of the tropics such data are absent, incomplete or unreliable. Despite their high costs, biodiversity surveys remain critical to achieving the effective allocation of conservation resources (Balmford and Gaston 1999; Gardner and others 2008). In practice, such surveys remain prohibitively demanding in terms of expertise, time and costs. Here, we consider if alternative approaches, that make better use of local knowledge, might offer a useful more cost-effective to managers.

A considerable area of the world’s tropical forests lie outside of strictly protected areas. Much of this land falls in timber concessions and other areas under the responsibility of local managers. For example, worldwide it is estimated that 1.2 billion ha of forest lies in production areas. This area is almost four times as large as the global area designated for stricter forms of protection (FAO 2010). In the species rich wet tropics, timber production forests cover more of the remaining natural forest area than do more strictly protected areas (Blaser and others 2011). Numerous other forest areas are managed as mineral concessions, and other commercial ventures (Meijaard and Sheil 2012). Such forests areas will not remain wholly pristine, but if well managed, they can greatly augment the conservation value of larger forested landscapes (Meijaard and Sheil 2007). Ensuring that managers can protect the environmental and biological values of these areas has become a major preoccupation of certification bodies and others seeking to maintain global biodiversity (Sheil and others 2010; Colchester and others 2009; FSC 1994). To achieve such management requires knowledge on what species occur in what locations. A major limitation is the high cost of gathering useful data. Even in many strictly protected areas, resources are limited and managers need to prioritize their activities to achieve the maximum benefits.

Classic biodiversity surveys require trained taxonomists and other specialists able to employ demanding and sophisticated methods (Kati and others 2004). But local communities are increasingly encouraged to play a role in natural resource assessment, management, and planning through consultation, data collection and clarification in the tropics (Sheil and Lawrence 2004; Hellier and others 1999; Wang and others 2004). Here, we ask if local people can provide useful, reliable, information about species of conservation concern.

Malinau, East Kalimantan (Indonesian part of Borneo) is extraordinarily rich in biodiversity. The local people possess a deep knowledge of the natural resources, which includes thousands of plant and animal species, their uses and where they occur, in forested landscapes (Sheil 2002; Sheil and others 2003, 2006). The more accessible parts of this landscape have changed considerably over the last 10 years due to logging, mining, and plantation projects: the degradation, fragmentation, and loss of forest looks likely to continue. There is a widespread recognition amongst both locals and outsiders that good land use planning is required, and that this should involve good information on conservation targets (Padmanaba and Sheil 2007).

Until recently, local knowledge has not been used much in conservation assessments especially in Indonesia. Our study is relevant for managers and other decision makers such as concession owners, conservation area managers and auditors as our results illustrate how indigenous people’s knowledge can provide urgently needed data with little cost.

## Materials and Methods

### Study Area

Malinau District, comprising 4.2 million ha, more than 90 % of which remains forested, lies between 1°5′22″N to 4°7′48″N and 114°31′24″E and 116°51′9″E in north Eastern Kalimantan. The climate is tropically humid with an average annual rainfall of 4,000 mm. Dry periods are usually <2 months in duration. The upper Malinau is steep, rugged, and the soils are poor and prone to erosion (Basuki and Sheil 2005). The region has become increasingly subject to timber and mining concessions, crop planting development and road building projects especially since the district became autonomous following decentralization in 2001 (Moeliono and others 2009).

**Fig. 1:**
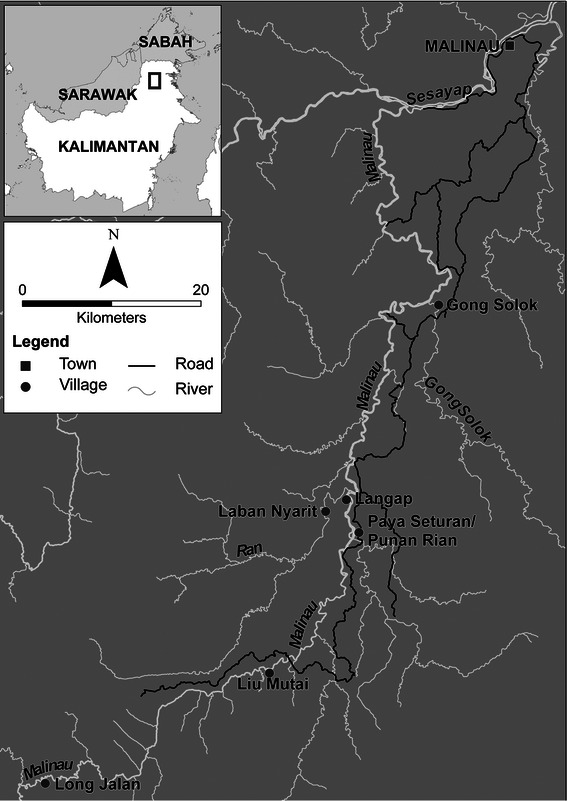
Study area in seven villages along the Malinau River. Sources: topography map (TOPDAM), road and Malinau village map (CIFOR)

Our study included seven villages in the Malinau watershed (Fig. 1) where Paya Seturan and Punan Rian are two administratively separate villages residing in the same settlement. All are dominated by two local ethnic groups i.e., the Merap and Punan. The Merap (Gong Solok, Langap, Paya Seturan villages) are mostly rice farmers and politically influential. The Punan (Punan Rian, Liu Mutai and Long Jalan villages), who engage mostly in extractive forest-based activities and limited agriculture, are less politically visible. Laban Nyarit Village consists of both Merap and Punan. Our seven study villages have a combined population of more than 1,700 and a density of <1 person km^−2^. Langap is the largest and Punan Rian the smallest (Table 1).

**Table 1:** The seven selected study villages in the Malinau watershed

Village	Location	Territory (km^2^)	Households	Population	Inhabitants (person km^−2^)
Gong Solok	3^o^19′19.319″N and 116^o^33′18.305″E	324	51	245	0.76
Paya Seturan	3^o^5′29.473″N and 116^o^28′29.858″E^a^	22^a^	28	157	12.00 ^a^
Punan Rian			25	107	
Langap	3^o^7′29.250″N and 116^o^27′43.792″E	469	131	666	1.42
Laban Nyarit	3^o^6′46.250″N and 116^o^26′28.975″E	256	50	237	0.93
Liu Mutai	3^o^56′56.877″N and 116^o^23′5.324″E	370	31	154	0.42
Long Jalan	2^o^50′14.066″N and 116^o^9′32.283″E	748	50	218	0.29

^a^A shared territory between two villages. In practice these villagers use a much larger area

### Methods

We selected two plants and six animals of regional conservation concern in Kalimantan (Table 2) and explored local recognition and observations of these species, including the habitats where they occur. We used colored high resolution images from illustrated books (Payne and others 2000; Puri 2001) and other pictures regarding the selected species.

**Table 2:** List of the eight selected species of conservation concern considered in our interviews with informants

Plant/animal species	Common name	Conservation status	Major threats
*Rafflesia* spp.	Rafflesia	Protected under the Republic of Indonesia’s Government Regulation 7/1999	Traditional medicines sellers, collectors, as well as habitat loss (CIFOR 2003).
*Coelogyne pandurata*	Black orchid	Protected under the Republic of Indonesia’s Government Regulation 7/1999	Illegal collection and forest fires (Puspitaningtyas and fatimah 1999)
*Helarctos malayanus*	Sun bear	Vulnerable (IUCN Red List) and Appendix I CITES	Forest conversion, logging activities, habitat fragmentation (Servheen 1999) and being hunted for medicines and food (Onuma and others 2001).
*Neofelis diardi/N. nebulosa*	Clouded leopard	Vulnerable (IUCN Red List) and Appendix I CITES	Deforestation (Rabinowitz and others 1987), habitat destruction and poaching (Brown and others 1995).
*Tarsius bancanus*	Tarsier	Vulnerable (IUCN Red List) and Appendix II CITES	Logging (Meijaard and others 2005), habitat destruction (Gursky and others 2008).
*Nasalis larvatus*	Proboscis monkey	Endangered (IUCN Red List) and Appendix I CITES	Habitat conversion and hunting (Meijaard and Nijman 2000).
*Nycticebus coucang*	Slow loris	Vulnerable (IUCN Red List) and Appendix I CITES	Habitat loss, hunting (Meijaard and others 2005) and pet trade (Fitch-Snyder and Schulze 2001).
*Pongo pygmaeus*	Orang-utan	Endangered (IUCN Red List) and Appendix I CITES	Hunting and pet trade (Meijaard and others 2005) as well as habitat loss through forest fires and human activities

We had previously worked with each community in 1999–2000 to develop geographically referenced maps as part of our participatory study. The making of these maps is documented in Sheil and others (2003). These involved the production of large-scale base maps with geo-referenced features (rivers, river junctions, roads, settlements and mountain peaks) which were cross-checked and labeled with names and developed further with a few local informants (river names, old village sites etc.) in each community. These base maps were then the basis of joint “freehand” mapping activities involving mixed groups of men and women in introductory community meetings in each of the seven villages—these maps were then combined, refined and checked (through field work) for the following month of project activities in each community. Mutual trust and collaboration have been developed between the Center for International Forestry Research (CIFOR) researchers and these local communities since that time. In this fieldwork, we used these maps, books and pictures, as references during interviews with informants, in November 2007 and January 2008.

In each of the seven villages, we interviewed informants, either individually or in groups, who were recognized within their community for their knowledge on the forest and its resources. Recognizing the time required we offered a small payment as an incentive to participate (USD 5.5 per day) with most informants giving 2–3 h for each interview. In total, 52 informants contributed information. All were men over 20 years old who regularly hunted and collected products in the forests. Each informant possessed experience regarding the forest and its resources and was staying in the village at that time. Women in our study villages rarely went to the forest; they worked mostly in their agricultural fields and gardens. Compared to men, they had less knowledge about distant locations and so after some discussion we decided not to include them in our interviews.

As we were concerned about the accuracy of our respondents’ memories, we specifically asked them to remember encounters within the last 10 years. We used our first visit to their villages in 1999 or 2000, where we (generally 10–12 researchers) stayed for a month while working with each community, as a shared reference point. For summary purposes, locations where the species were observed were also classified into three major land types or “habitats” i.e., forest, village, and agricultural field. Village was defined as the land immediately around the settlement while agricultural field included all croplands, open fallow, and plantations.

We asked when and in which habitat people had detected each of the eight selected species, whether the observations were direct or indirect (e.g., calls, prints, dung, and marks). Respondents indicated on the map where each observation had occurred. In the case of direct sightings, we requested further details including time of day and the number of individuals seen. We also enquired how familiar people were with each species, and any uses they had, and how certain they were that they could identify them.

From the published literature, we gathered information that might contradict or verify the information received from our respondents. We summarized the cost of our activities in terms of time and expense and compared this to the cost of similar survey activities conducted entirely by professional scientists without local guidance or input.

## Results

### What, Where, and When?

**Fig. 2:**
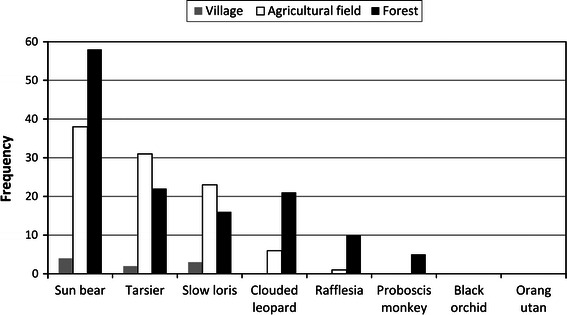
Number of selected species observed by local people in different land types

Among our eight study species, the sun bear, tarsier and slow loris were the most frequently noted by our informants (Table 3; Fig. 2).

**Table 3:** Records of the selected eight species of conservation concern seen during the previous 10 years and reported by informants from the seven villages

Plant/animal	Village (# respondent)						
	GS (11)	Lg (7)	LN (5)	PS (9)	PR (5)	LM (8)	LJ (7)
Sun bear	26	17	28	6	7	6	10
Tarsier	16	7	22	1	5	1	3
Slow loris	10	9	11	2	4	3	3
Clouded leopard	9	4	5	1	3	1	4
Rafflesia	2	0	0	1	3	0	5
Proboscis monkey	1	2	0	0	0	0	2
Black orchid	0	0	0	0	0	0	0
Orang-utan	0	0	0	0	0	0	0

Note: *GS* Gong Solok, *Lg* Langap, *LN* Laban Nyarit, *PS* Paya Seturan, *PR* Punan Rian, *LM* Liu Mutai, *LJ* Long Jalan

The sun bear was seen at any time of day in all land types but was mostly observed in the forest. Two respondents in Gong Solok and two others, one in Langap and one in Long Jalan, reported seeing sun bear near their village. Respondents considered the sun bear a solitary animal though a mother was sometimes seen with a cub. However, in one observation, six animals were seen eating durian (*Durio* sp.) fruit in riparian forest near Gong Solok.

Local people noted the presence of the sun bear from its nest which was built in preferred trees such as *Ochanostachys amentacea, Lithocarpus cantleyanus* and *Shorea parvifolia*. They were also reported based on their calls, footprints, and distinctive claw marks on trees. According to the informants, the bears are often associated with fruiting trees such as *Durio* sp., *Nephelium* *ramboutan-ake* and *Dimocarpus longan*.

The sun bear was the only species in our list seen as having a significant value for either use or trade: its gall bladder has ‘medicinal value’ and other parts including skin, claws and teeth are used as ornaments. The Punan will consume bear meat, but it is taboo for Merap. Our respondents volunteered various reasons for killing sun bear including self-defense, for the ornament trade; and sometimes to keep the cubs as pets.

Tarsier and slow loris were observed mostly at night in agricultural fields (Fig. 2), where they are associated with shrubs and trees. Tarsier and slow loris are considered solitary. Though neither species is actively sought, slow loris are occasionally kept as pets.

Clouded leopard (*Neofelis diardi/N. nebulosa*) was known to informants from all seven villages, but was infrequently seen. Informants explained that it is hard to see because this nocturnal animal mostly lives in dense forest and is wary of people. Clouded leopards were also occasionally killed for their claws, teeth and skins, which are used and sold as ornamental items.

The giant flowers of the parasitic plant rafflesia (*Rafflesia* spp.) were seldom encountered. It was easily recognized when flowering, usually during the rainy season. An informant in Gong Solok who went for hunting in the forest saw a flowering rafflesia and said it smelled like a decomposing animal and there were many flies around it. The flower had decayed when he passed it three days later. There were no records for black orchid (*Coelogyne pandurata*) and no sighting of orang-utan (*Pongo pygmaeus*) from within the last decade (but see later). Rafflesia, black orchid and orang-utan had no reported use.

Two respondents each in Langap and Long Jalan had observed proboscis monkey (*Nasalis larvatus*) in the forest. The villagers were familiar with the species as it was frequently seen in the mangrove forests along the lower Sesayap River when traveling by boat on the commonly used route from Malinau to Tarakan city on the coast. The authors have also seen these animals frequently on the same route. Those who had seen the proboscis monkey in the forest also explained that they would hunt it for food.

**Fig. 3:**
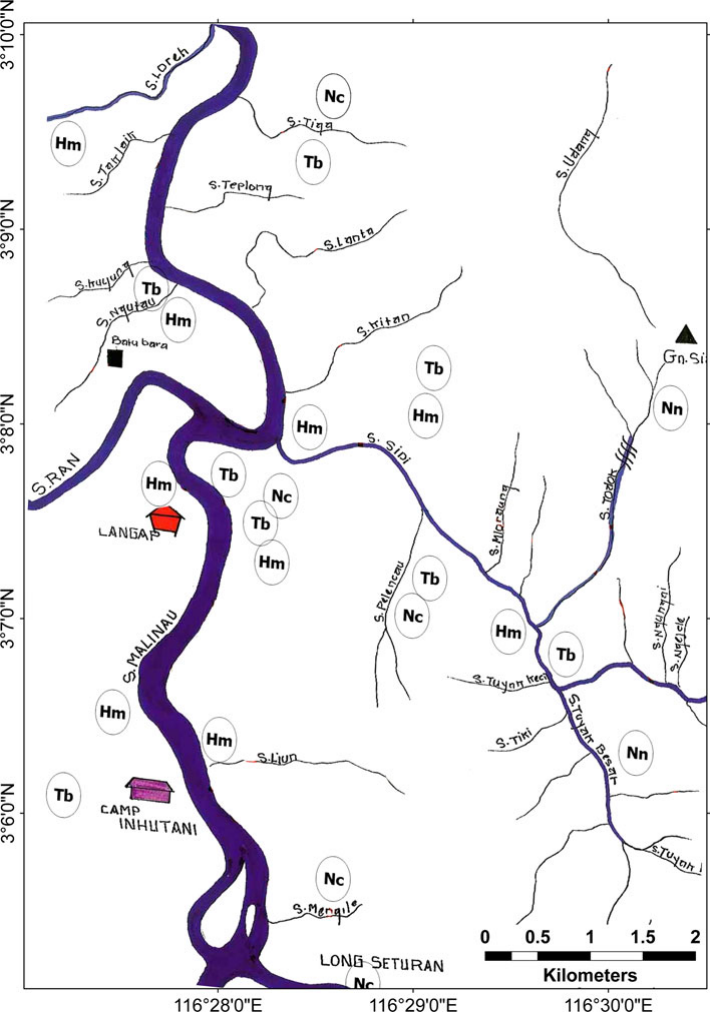
A portion of the sketch map of Langap Village

**Fig. 4:**
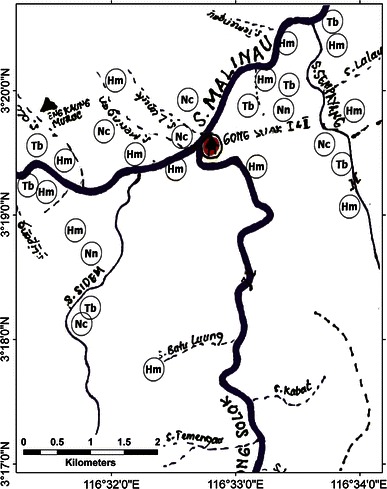
A sketch map of Gong Solok Village

**Fig. 5:**
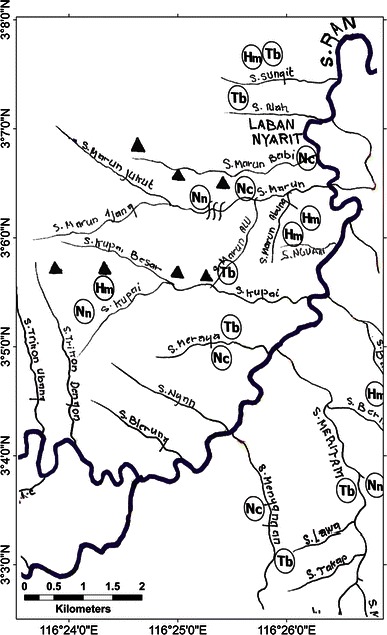
A sketch map of Laban Nyarit Village

**Fig. 6:**
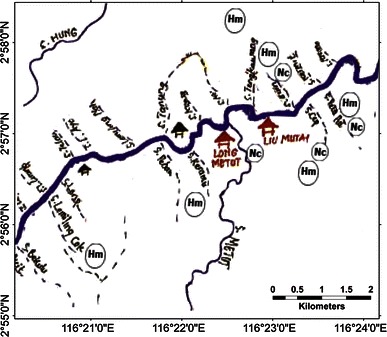
A sketch map of Liu Mutai Village

**Fig. 7:**
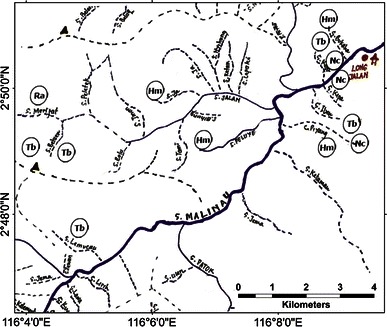
A sketch map of Long Jalan Village

**Fig. 8:**
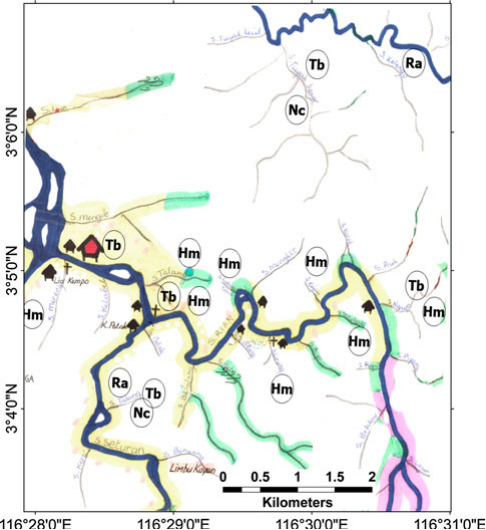
A sketch map of Paya Seturan and Punan Rian villages

A sketch map of Langap village showing where the selected species occur, with markers indicating approximate position for each sighting is provided in Fig. 3. Sun bear, tarsier and slow loris were widely distributed in the village’s territory and recorded even in agricultural fields near the settlements. Most clouded leopard and rafflesia sightings occurred in the forest distant from the village. We provide complete maps for the other six villages in the appendix (Figs. 4, 5, 6, 7, 8).

In addition to the local records from the previous 10 years presented in Table 3, some informants offered encounters that were older or from other regions. An elderly informant in Gong Solok had seen orang-utan in hill forest at the upper part of Temengau River (one branch of Gong Solok River) in the 1970s. In addition one record of proboscis monkey and one of orang-utan came from informants who had seen them in the Kayan and Bahau watersheds respectively (outside the range of the maps we were working with).

### Costs

The cost for 1999–2000 mapping activities was 420 USD approximately to cover specific fee for informant in the seven villages, but here we exclude this original mapping cost and focus on the costs for the more recent eight species study. The field portion of our surveys with the seven villages required one graduate researcher for 6 weeks. The total cost was less than USD 5 200. The main expenses were the researcher’s salary (USD 3 250), informant expenses (USD 274), local transport (car and boat USD 628; a car cost USD 75 including driver, fuel being an additional cost, for the three upstream communities boats provided the only access and cost USD 220 plus gasoline) and accommodation (USD 421). The other miscellaneous costs include food (USD 368) and equipment including paper, books, maps and printing (pictures), etc. (USD 179). The time to compile and assess these data involved another two days for the researcher to update the reference maps.

## Discussion

The informants were familiar with seven of our eight study species; that is all except the black orchid. People even considered sun bear, slow loris and tarsier relatively common. These results indicate that the region hosts populations of species of recognized conservation significance. Clearly, forest loss in this region was already a conservation concern but with local knowledge we now have a better idea of some species to consider more explicitly in planning. But before we start discussing such conservation, we need to consider if our data are credible. We briefly review the evidence.

### Is Information from Local People Reliable?

One recent study around the Seturan River in Malinau estimated, based on camera traps and animal signs, that there is ~1 sun bear/24 km^2^ in the primary forest areas (Augeri 2005). While they could not offer values our informants believe the density of sun bears is low although the animals are widespread. Our own observations confirm the presence of bears. We often saw distinctive scratch marks on trees, prints in mud, young animals kept as pets, and even occasional direct sightings (e.g., Imam Basuki personal observation 2003). We conclude that Malinau still maintains a notable population of this species.

Tarsiers occur in western Indonesia (including Sulawesi) and Philippines (Gorog and Sinaga 2008). Though we ourselves never saw these animals, we find the reports from our informants in Malinau credible. Local knowledge concerning tarsiers, i.e. their body size, habitat preference and behavior matched published accounts (Roberts 1994; Crompton and Andau 1987). Local observations appear a credible means to clarify tarsier distributions.

Slow loris are known to occur in primary and secondary forests in South and South East Asia including Borneo (Meijaard and others 2005). Nonetheless, we know little about its regional status and the impact of hunting (especially for the pet trade). According to our informants, the species remains widespread in Malinau suggesting that this region hosts a major population. As with the tarsiers, we find the reports credible, with descriptions, including its slow movements, matching confirmed accounts.

Clouded leopards occur in tropical rain forests from Nepal and southeastern China, through Thailand, peninsular Malaysia, to Sumatra and Borneo (Azlan and Lading 2006). Some experts consider the Borneo Clouded leopard (*Neofelis diardi*) distinct from the Asia mainland species (*Neofelis nebulosa*) increasing its conservation significance (Kitchener and others 2006; Christiansen 2008). Published records are few. The species is wary of humans and seldom seen. Although widely reported in Malinau, our informants seldom encountered it directly. Nonetheless, we have no doubt about the reliability of these local reports. During our study, we saw two dried skins of clouded leopard shot in 2008, one each in Paya Seturan and Gong Solok.

Proboscis monkeys are endemic to Borneo. They are usually considered mangrove specialists, but are known to sometimes disperse into the headwaters of major rivers (Meijaard and Nijman 2000). Most published accounts consider studies conducted in Sabah and Sarawak (Boonratana 2000) and little is known about this species in northeastern Kalimantan. One respondent in Long Jalan said he had killed a proboscis monkey in the forest near the village in 2004. Currently, the natural habitat of the monkey is the tidal swamps along the lower Sesayap River, about 100 km to the north of Long Jalan. The records in Malinau were likely to be rare observations of dispersing individuals. Our informants have contributed significant information on the distribution and dispersal behavior of proboscis monkeys in Malinau.

The total population of orang-utan in 1997 in East Kalimantan was more than 4,200, but none have been reported in Malinau (Rijksen and Meijaard 1999). Our study found no records over the last 10 years. From Rijksen and Meijaard (1999) data and our own lack of observations, we consider the report from our informants credible. The existence of the older observations suggests that the species has become recently locally extinct, or that individual animals may occasionally disperse from neighboring regions. Both ideas deserve further study as the status of this species is a topic of major conservation concern (Bernard and others 2002; Wich and others 2008). For example, the knowledge that Malinau historically hosted orang-utan populations would suggest that this area is suitable for reintroduction.

Rafflesia has seldom been reported in East Kalimantan. Until 2003, only two well-documented records had come from Kalimantan with other accounts referring to the genus in Malaysia and Brunei (Sheil quoted in CIFOR 2004). Recently, three additional records were added from Malinau district: one recorded by researchers working at the CIFOR camp near Seturan River with photographic evidence (likely to be *R. pricei*), another was seen by a CIFOR researcher near the Tubu (Edmond Dounias personal observation 2003), and yet another was seen in the joint expedition on biodiversity in Kayan Mentarang National Park, coordinated by the World Wide Fund for Nature (WWF) Indonesia, in 2003. Now 11 distinct records of rafflesia have been added by respondents from four of our seven study villages. If we accept these observations, this greatly extends our knowledge of the distribution of this seldom-reported genus in Kalimantan. It seems unlikely that our informants could confuse rafflesia with anything else as its distinctive flower has a well defined form, coloring and odor. Malinau appears to host a considerable population of these remarkable plants.

Black orchids occur in Sumatra, Peninsular Malaysia, Borneo and possibly the Philippines (Sierra and others 2000). It typically inhabits heath land and sandy quartz areas with peat (Puspitaningtyas and Fatimah 1999). There have been no reports of black orchid in Malinau. All our informants replied in the negative when asked about the occurrence of this plant. A completely negative result also helps to reassure skeptics that informants do not invest in supplying us with positive observations just to ‘keep us happy’. The chance of all 52 respondents failing to recognize this species, if there was a tendency to fabricate answers, is very low. Indeed, we would suggest that all surveys of this nature include one or more species believed not to occur in the region. This could act as a “fabrication check”—this concern becomes greater when informants are paid and may feel obliged to invent answers rather than to disappoint. Our informants have passed this check implying that the information provided appears unlikely to be fabricated.

It is well beyond our research budget to conduct field examinations to judge the accuracy on all the species information, but the above discussion suggest that information from our respondents reflects a rigorous system of knowledge concerning local flora and fauna. Our study thus adds a further case attesting to the credibility of local knowledge (Traditional Ecological Knowledge) as has been indicated elsewhere, e.g. in Canada (Kowalchuk and Kuhn 2012), Africa (Domfeh 2007), China (Wang and others 2004).

### Challenges Regarding Wider Application

Our results depend upon our respondents’ experiences and memories. If this method was to be applied more widely, several practical issues would need consideration: who to work with, how to ensure effective communication, deciding what to believe, and avoiding cultural obstacles and misunderstandings (Sheil and Lawrence 2004). In this study, our informants seemed to be knowledgeable and willing to share their information. Importantly, the reliability of the observations seems high, and we are not aware of having had any significant cultural misunderstandings. In other circumstances, the approach may be less effective as people may know less, be less willing to share, might wish to mislead or may not provide reliable information for any one of a variety of reasons. In Malinau, we have established a good relationship with these communities, and this contributes to trust and a willingness to share knowledge that cannot be taken for granted.

The accuracy and coverage of local observations are of interest. The implied accuracy of the placement on the map is not high though in most cases conservation activities would not need precision. If greater accuracy was needed, we would advocate a specific visit with a GPS to achieve this—the informants would guide, or could record the location themselves. In technical surveys, there is usually some effort to distribute sampling effort to achieve good coverage—with local informants this is not possible. In future cases, we could ask each informant about which areas they had visited with what frequency and to use this to characterize coverage in terms of observation “effort” or “intensity.”

### Opportunities and Applications

Other commentators and studies have shown how local knowledge can increase the effectiveness of management decisions when integrated with the scientific knowledge (Charnley and others 2007; Barrios and others 2006; Sobrevila 2008). The value and reliability of local knowledge has been noted in many studies, and dialogue between local and scientific knowledge has been seen to lead to improved resource management (Barrios and others 2006; Rist and Dahdouh-Guebas 2006).

In many regions of the world, there is an urgent and specific need for effective methods that can help managers plan and make better decisions (Kati and others 2004). Several studies have highlighted the cost-effectiveness of performing biodiversity surveys as an input to ensure the effective allocation of resources (Balmford and Gaston 1999; Gardner and others 2008). While such technical surveys may pay for themselves in terms of long-term conservation benefits, it is less certain what should be done in areas where we need information soon, but the necessary resources and expertise for technical surveys are unavailable. Our study bolsters our assertion that engaging local knowledge in biodiversity surveys can be not only cheap and practical, but also provide valuable support to achieve conservation outcomes.

Involving the local community in biodiversity assessment offers a simple short cut to clarify the presence and distribution of conservation target species in any area where limited resources for conservation are a constraint. Our study of eight species in seven villages took one and a half months and cost less than USD 5200. Most of the costs are in transport, logistics, and time: assessing additional species would have added very little to costs. It is hard to find comparable figures in terms of the aims of a technical study required to achieve similar objectives. If we had tried to directly map the various species in the village territories by direct observations, signs and other technical methods (such as camera traps), we estimate additional costs due to additional (a) expertise (b) time and (c) equipment and logistics, would be greater than in the present study by at least an order of magnitude at around 150,000–400,000 USD. There would be some benefits: the data would be collected in a systematic fashion and taxonomic precision could be better guaranteed. But even in extensive expert surveys, coverage would inevitably remain incomplete and elusive species would remain poorly documented. We suggest that for elusive species such as rafflesia and clouded leopard working through local people will remain not only a cheaper but also a more effective survey method than technical approaches.

In addition to budget and time, efficient local participation can help legitimize conservation activities by managers. Moreover, when properly designed, the results may yield just as relevant results as those generated from professional surveys and applicable not only at the local level, but also at regional and global levels (as noted in the context of monitoring by Danielsen and others 2005). Certainly, we need to be able to trust the information gathered. Some ground-truthing of results may often be desirable, especially when results are surprising, or when costly decisions are to be made based on the results, but local knowledge and information can play a major role in making the process more targeted and cost-effective.

Local informants may not always be reliable. In general, we believed that informants were well able to recognize and report the species and information we were asking for, but people sometimes had difficulty in remembering date and time. To address this, we limited our discussion in the last 10 years of observation. Overall we are confident that the approach is applicable to species which are distinctive and locally known. When species are hard to observe, difficult to identify and distinguish, or attract little attention, local informants may be less aware. Certainly, we would need to consider such issues of apparency when applying such an approach more generally—and we note that this will also reflect the communities being questioned (Sheil and Salim 2012). In general, people are willing to express their doubts and limitations. When people express doubt or when results are inconsistent other approaches may be necessary. Reliance on local informants may also lead to issues of credibility—for example the disputed presence of an unexpected species—that cannot readily be addressed without formal verification via an alternative more technically formalized survey approach. This may lead to additional costs. In some cases local people may still be able to help, e.g., by leading the external experts to the location of the species, collecting botanical specimens or showing where camera traps should be installed. In other cases, more expensive surveys may be unavoidable. However, by raising such questions for scrutiny only in specific cases, the use of local informants still offers a more cost-effective approach that would less-focused surveys.

Local knowledge-based surveys are a sensible approach to conduct low cost assessments of conservation values and significance across much of the tropics. Not only they are much cheaper than expert dependent methods, they are also relatively quick and simple. These approaches could readily be adopted by managers, local land use planners, and those who contribute to conservation processes. The ongoing development and application of “High Conservation Values” concepts (Dennis and others 2008) could also be facilitated by the systematic incorporation of local knowledge and preferences.

## Conclusions

Extensive and reliable conservation surveys provide a basis for sound conservation planning, and conservation friendly management practices. Such surveys are lacking in many parts of the world, including Indonesian Borneo where the expertise and resources available pose severe constraints. If we wait for extensive expert-led surveys, many forests will be degraded or lost before their conservation values have been even partially evaluated. Local knowledge and participation facilitates effective low cost conservation surveys. We recommend that managers make better use of such potential collaborations. While constructive, such involvement is only a first step in better engaging local people in improved management—but as it offers direct benefits to conservation outcomes, it is one that can and should be widely promoted. From such beginnings, we can hope for deeper collaborations that make use not only of knowledge but also of preferences. Building dialogue between local communities and managers can be relatively cheap and easy, and can create new opportunities for improved management outcomes.

## Appendix

See Figs. 4, 5, 6, 7, 8.
